# Supplementary material for: Reconciling Mining with the Conservation of Cave Biodiversity: A Quantitative Baseline to Help Establish Conservation Priorities
Source: PLoS One. 2016 Dec 20;11(12):e0168348. doi: 10.1371/journal.pone.0168348 (PMC5173368; doi:10.1371/journal.pone.0168348)
Supplement: S1 Dataset — (ZIP) [file pone.0168348.s002.zip › Taxa/Serra Norte/SN_2013/N4WS/Lista N4WS-42.pdf]

CAVIDADE N4WS-0042

Classe	Ordem	Fam/Outros	Gên/Outros	Espécie	S-U	U-S	Zon.
Arachnida	Acari	Neothyridae	*Diplothyrus*	*schubarti*	X		P
Arachnida	Amblypygi	Phrynidae	*Heterophrynus*	*longicornis*	2		E/P
Arachnida	Araneae	Drymusidae		jovem	X		E
Arachnida	Araneae	Ochyroceratidae		jovem	X		E
Arachnida	Araneae	Salticidae		sp.2	X	X	P
Arachnida	Araneae	Theridiosomatidae	*Plato*	sp.1		X	P
Arachnida	Opiliones	Sclerosomatidae		jovem	X		E
Arachnida	Pseudoscorpiones	Chernetidae	*Spelaeochernes*	sp.1	X	X	E/P
Arachnida	Pseudoscorpiones	Chthoniidae	*Pseudochthonius*	sp.1	X		E
Chilopoda	Geophilomorpha	Ballophilidae	*Ityphilus*	sp.1	1		E
Diplopoda	Polydesmida	Pyrgodesmidae		sp.1		X	P
Entognatha	Diplura	Campodeidae		sp.1		X	P
Insecta	Blattodea			jovem	1		E
Insecta	Coleoptera	Staphylinidae		sp.66		X	E
Insecta	Diptera	Cecidomyiidae	Cecidomyiinae	sp.	X		E
Insecta	Diptera	Psychodidae	*Sciopemyia*	*sordellii*	X		P
Insecta	Diptera	Tipulidae	Tipulinae	sp.	X		E
Insecta	Hymenoptera	Formicidae	*Nylanderia*	sp.1	X		E/P
Insecta	Orthoptera	Phalangopsidae	*Paraclodes*	sp.	2	1	E
Insecta	Orthoptera	Phalangopsidae	*Phalangopsis*	sp.	21	12	E
Gastropoda	Pulmonata	Systrophiidae	*Happia*	sp.	X		E
Amphibia	Anura	Strabomantidae	*Pristimantis*	cf. *fenestratus*	1		E
